# Stochastic modelling of cellulose hydrolysis with Gauss and Weibull distributed transition probabilities

**DOI:** 10.1038/s41598-021-88873-6

**Published:** 2021-05-04

**Authors:** Joseph Mcgreg Duru, Oana Cristina Pârvulescu, Tănase Dobre, Cristian Eugen Răducanu

**Affiliations:** Chemical and Biochemical Engineering Department, University POLITEHNICA of Bucharest, 1-6 Gheorghe Polizu, 011061 Bucharest, Romania

**Keywords:** Engineering, Mathematics and computing

## Abstract

Two Markov-type stochastic models were developed to describe the kinetics of acid hydrolysis of cellulose. One of them involved a Gauss (normal) distribution of probabilities of chemical bond breaking, the other a Weibull distribution. It was considered that the random breaking of cellulose was based on the cleavage of a parent macromolecule into two descendants. Model equations and kinetics of acid hydrolysis of cellulose consisting of 10 and 100 units of cellobiose were presented. The effects of acid concentration and temperature on the kinetics of hydrolysis process were taken into account. The results obtained applying both stochastic models were in a reasonable agreement with those obtained using a deterministic kinetic model. These stochastic models can accurately describe the kinetics of acid hydrolysis and cover the drawbacks of some deterministic kinetic models, e.g., large number of model equations and parameters, modification of parameter values by changing the process conditions.

## Introduction

Thermal or (bio)chemical degradation of biopolymers, e.g., cellulose, hemicellulose, starch, lignin, other low and medium molecular mass polysaccharides, has been studied extensively for the last twenty years^1–10^. Chemical or enzymatic depolymerization of cellulose or starch occurs in aqueous suspension and is known as hydrolysis. Despite of the fact that the cellulose or starch hydrolysis is a heterogeneous (bio)chemical process, it is often analyzed using formal kinetics.

The first models describing dilute acid hydrolysis of cellulose were based on pseudo-homogeneous irreversible first-order reactions, e.g., those presented in scheme (1), where *k*_1_ and *k*_2_ are kinetic constants^11–15^. According to scheme (1), time (*τ*) variation of glucose concentration (*c*_*G*_) can be expressed by Eq. (2), where *c*_*C*0_ is the initial cellulose concentration.

1 $${\text{Cellulose }}\left( {\text{C}} \right)\mathop{\longrightarrow}\limits^{{k_{1} }}{\text{Glucose }}\left( {\text{G}} \right)\mathop{\longrightarrow}\limits^{{k_{2} }}{\text{Degradation }}\;{\text{products}}$$

2 $$c_{G} \left( \tau \right) = c_{C0} \left( {\frac{{k_{1} }}{{k_{1} - k_{2} }}} \right)\left( {e^{{ - k_{1} \tau }} - e^{{ - k_{2} \tau }} } \right)$$

In the case of enzymatic hydrolysis, three basic processes, which are shown in scheme (3), where *k*_1_, *k*_2_, and *k*_3_ are kinetic constants, whereas *E*1 and *E*2 are specific enzymes, can be assumed. Three types of enzymes are commonly considered in the biochemical hydrolysis of cellulose, i.e., endocellulases, exocellulases, and cellobiases (β-glucosidases). The mode of action of these enzymes is as follows: (i) endocellulases break cellulose chains into shorter polysaccharide chains; (ii) exocelullases attack from the ends of these shorter polysaccharide chains resulting in oligosaccharides, predominantly cellobiose; (iii) cellobiases cleave the cellobiose into glucose. In the presence of endo/exocellulases (*E*1) and β-glucosidases (*E*2), the processes presented in scheme (3) can be described by simple first-order kinetics expressed by Eqs. (4)−(6), where *c*_*C*_ and *c*_*CB*_ are concentrations of cellulose and cellobiose in the reaction medium, *v*_*R*1_, *v*_*R*2_, and *v*_*R*3_ are reaction rates. Reaction rate constants for the first and third process depend on the adsorption kinetics of enzymes on cellulose chains^16–18^. Assuming a rapid adsorption of enzymes *E*1, the process occurs near to equilibrium. Accordingly, considering a Langmuir adsorption isotherm, *k*_1_ and *k*_3_ are given by Eqs. (7) and (8), where *c*_*E*1_ is the concentration of enzymes *E*1 in the reaction medium, *K*_*L*_ represents the Langmuir constant for sorption equilibrium, *k*_max1_ and *k*_max3_ are the maximum values of *k*_1_ and *k*_3_ at full saturation of the substrate with enzymes *E*1 for the first and third process in the scheme (3)^19^. To determine the reaction rate constant for the second process (transformation of cellobiose into glucose), the activity and concentration of enzymes *E*2 in liquid phase as well as the fact that there is a classic enzyme catalysis described by Michaelis–Menten equation must be taken into account^19^. Moreover, the effect of process temperature, reaction inhibition by product and substrate should be considered^18–23^.

3 $${\text{Cellulose }}\left( {\text{C}} \right)\mathop{\longrightarrow}\limits^{{k_{1} (E1)}}{\text{Cellobiose }}\left( {{\text{CB}}} \right);{\text{ CB}}\mathop{\longrightarrow}\limits^{{k_{2} (E2)}}{\text{Glucose }}\left( {\text{G}} \right);{\text{ C}}\mathop{\longrightarrow}\limits^{{k_{3} (E1)}}{\text{G}}$$

4 $$v_{R1} = k_{1} c_{C}$$

5 $$v_{R2} = k_{2} c_{CB}$$

6 $$v_{R3} = k_{3} c_{C}$$

7 $$k_{1} = \frac{{k_{\max 1} c_{E1} }}{{K_{L} + c_{E1} }}$$

8 $$k_{3} = \frac{{k_{\max 3} c_{E1} }}{{K_{L} + c_{E1} }}$$

The decomposition of (bio)polymers leads to a large number of components involved in complex schemes of parallel and consecutive reactions. For example, in the thermal degradation of halogenated polymers, i.e., poly(chloroprene) and poly(vinyl chloride), kinetic mechanisms taking into account 38–40 species and pseudo-components (molecules and radicals) involved in 190–250 chemical reactions were presented^24,25^. It is very difficult in this case to apply mathematical models based on formal kinetics. Models assuming random scission of linear polymer chains^26^ and chain-end scission^27^ can be more appropriate. Mechanisms based on systematic breakage of polymer chains explain quite well the hydrolysis process of cellulose^28,29^. Markov chain was used to describe the enzymatic and acid hydrolysis of cellulose^30^. Markov-type stochastic models are commonly applied in all engineering fields where elementary processes with random evolution appear^31–33^.

Stochastic models of cellulose hydrolysis, which are based on two basic phenomena, i.e., breaking or non-breaking of polymer chain, could be effective and more realistic approaches. On the one hand, the concentration of cellulose-derived products and frequency distribution of molecular chain length during hydrolysis can be easily determined by a stochastic model, which can provide a better understanding of the mechanisms involved in the process. On the other hand, working with large transition probability matrices, imposed by the initial length of cellulose molecular chain or by the existence of several initial chain lengths, can be computationally quite complex.

Assuming that the highest probability of bond breaking is in the middle of the polymer chain, the transition probabilities can be expressed using a Gauss (normal distribution). A Weibull distribution can be applied if the breaking is more likely to occur at the ends of the polymer chain. For example, in the biochemical hydrolysis of cellulose, it can be assumed that endocellulases act in the middle of the cellulose chain, whereas exocelullases attack from the ends of polymer chains.

This paper aimed at studying the acid hydrolysis of cellulose. A Markov-type stochastic model was developed, assuming that the probabilities of chemical bond breaking followed either a Gauss (normal) distribution or a Weibull distribution.

## Methods

The stochastic model focuses on partial hydrolysates obtained by acid hydrolysis of cellulose, which are numerically characterized by their molecular masses. The basic assumptions of the model are as follows: (i) cellulose subjected to acid hydrolysis contains a finite number of hydrolysable polysaccharide species (cellulose fragments with different polymerization degrees) with known molecular masses (*M*_0_^*s*^, *s* = 1, 2*…S*) and concentrations; (ii) random breaking of a polysaccharide into several macromolecules is based on the cleavage of a parent macromolecule into two descendants; (iii) breaking of the polysaccharide chain into different fragments can occur with different probabilities; (iv) the continuous distribution of molecular masses of hydrolysates can be divided into discrete intervals with a molecular mass corresponding to each interval; (v) a hydrolysate within a molecular mass range (class) is divided into two macromolecules within lower molecular mass ranges; (vi) the process of molecular fragmentation by hydrolysis is a homogeneous Markov process; (vii) hydrolysates cannot participate in any coupling reactions.

**Figure 1:**
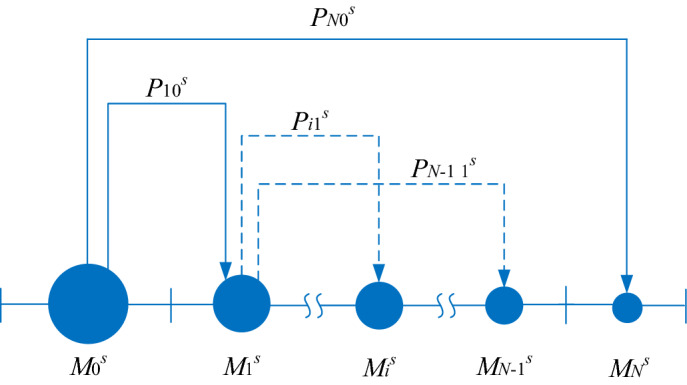
Stochastic model principle in terms of hydrolysate molecular mass intervals and transition probabilities for a polysaccharide species (*s* = 1, 2*…S*).

Stochastic model principle of hydrolysis of a polysaccharide species (*s* = 1, 2*…S*) is shown in Fig. 1. The breaking of a macromolecule within *M*_0_^*s*^ molecular mass class into one within *M*_1_^*s*^ molecular mass class and another within *M*_*N*_^*s*^ molecular mass class is represented with continuous line. The process evolution, *i.e*., the macromolecule within *M*_1_^*s*^ molecular mass class splits into one within *M*_*i*_^*s*^ molecular mass class and another within *M*_*N*−1_^*s*^ molecular mass class, appears with dotted line. Transition probability is denoted by *P*_*ab*_^*s*^, where the subscripts *a* and *b* refer to the molecular mass ranges of hydrolysates after and before breaking, respectively.

The mass balance for *s* species parent macromolecules within the molecular mass class *M*_*k*_^*s*^ which break at time *τ* into descendants within the molecular mass classes *M*_*l*_^*s*^ and *M*_*m*_^*s*^ is given by Eq. (9), where *m*_*k*_^*s*^ represents the mass of parent macromolecules, whereas *m*_*l*_^*s*^ and *m*_*m*_^*s*^ are the masses of descendants. Moreover, if a parent macromolecule breaks into two descendants, the probability of its breaking by hydrolysis (*p*_*k*_^*s*^) is equal to the probability of birth of each descendant (*P*_*lk*_^*s*^ and *P*_*mk*_^*s*^), as shown by Eq. (10).

9 $$m_{k}^{{\begin{array}{*{20}c} {} \\ \end{array} s}} = m_{l}^{{\begin{array}{*{20}c} {} \\ \end{array} s}} + m_{m}^{{\begin{array}{*{20}c} {} \\ \end{array} s}}$$

10 $$p_{k}^{{\begin{array}{*{20}c} {} \\ \end{array} s}} = P_{lk}^{{\begin{array}{*{20}c} {} \\ \end{array} s}} = P_{mk}^{{\begin{array}{*{20}c} {} \\ \end{array} s}}$$

For a short time interval (Δ*τ*), breaking probability of macromolecules within the molecular mass class *M*_*i*_^*s*^, *p*_*i*_^*s*^ (*i* = 0, 1…*N*), and breaking missing probability associated with this class, *P*_*ii*_^*s*^, are defined by Eqs. (11) and (12). Assuming that the hydrolysis of *s* species is a Markov homogeneous stochastic process, the transition probability from the molecular mass class *M*_*i*_^*s*^ to *M*_*j*_^*s*^, *P*_*ji*_^*s*^ (*i*, *j* = 0, 1…*N*, *j* ≠ *i*), is defined by Eq. (13), where *α*_*ji*_^*s*^ is the transition probability frequency and Δ*τ* the time interval. The transition probability in mass units, *P*_*ji*_^*s,m*^, is given by Eq. (14) depending on molecular mass classes and transition frequencies. Unsteady state species mass balance led to Eq. (15), expressing time variation of mass fraction of *s* species within the molecular mass range *M*_*i*_^*s*^, *ω*_*i*_^*s*^.

11 $$p_{i}^{{\begin{array}{*{20}c} {} \\ \end{array} s}} = \sum\limits_{\begin{subarray}{l} j = 1 \\ j \ne i \end{subarray} }^{N} {P{}_{ji}^{{\begin{array}{*{20}c} {} \\ \end{array} s}} }$$

12 $$P_{ii}^{{\begin{array}{*{20}c} {} \\ \end{array} s}} = 1 - p_{i}^{{\begin{array}{*{20}c} {} \\ \end{array} s}} = 1 - \sum\limits_{\begin{subarray}{l} j = 1 \\ j \ne i \end{subarray} }^{N} {P{}_{ji}^{{\begin{array}{*{20}c} {} \\ \end{array} s}} }$$

13 $$P_{ji}^{{\begin{array}{*{20}c} {} \\ \end{array} s}} = \alpha_{ji}^{{\begin{array}{*{20}c} {} \\ \end{array} s}} \Delta \tau$$

14 $$P_{ji}^{{\begin{array}{*{20}c} {} \\ \end{array} s,m}} = \frac{{M_{j}^{{\begin{array}{*{20}c} {} \\ \end{array} s}} \alpha_{ji}^{{\begin{array}{*{20}c} {} \\ \end{array} s}} }}{{\sum\limits_{n = 1}^{N} {M_{n}^{{\begin{array}{*{20}c} {} \\ \end{array} s}} \alpha_{ni}^{{\begin{array}{*{20}c} {} \\ \end{array} s}} } }}$$

15 $$\frac{{d\omega_{i}^{{\begin{array}{*{20}c} {} \\ \end{array} s}} }}{d\tau } = \sum\limits_{\begin{subarray}{l} h = 0 \\ h \ne i \end{subarray} }^{N} {P_{ih}^{{\begin{array}{*{20}c} {} \\ \end{array} s,m}} } \omega_{h}^{{\begin{array}{*{20}c} {} \\ \end{array} s}} - \omega_{i}^{{\begin{array}{*{20}c} {} \\ \end{array} s}} \sum\limits_{\begin{subarray}{l} j = 1 \\ j \ne i \end{subarray} }^{N} {P_{ji}^{{\begin{array}{*{20}c} {} \\ \end{array} s,m}} }$$

**Figure 2:**
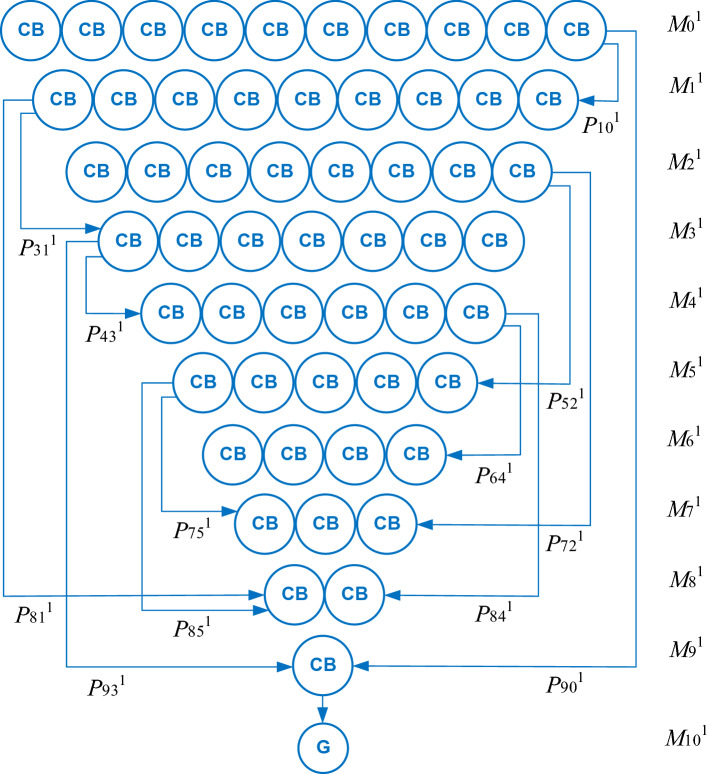
Hydrolysate molecular mass intervals and transition probabilities for a polysaccharide species (*s* = 1) consisting of 10 units of cellobiose (CB).

Current state of mass fractions of hydrolysable species within the molecular mass classes *M*_0_^1^, *M*_1_^1^…*M*_10_^1^ (Fig. 2) is given by the probability vector described by Eq. (16), where *τ*_*n*_, defined by Eq. (17), represents the current time and *n* is the number of time sequences taken into account in the evolution of hydrolysis process. Initial state of probability vector, which is expressed by Eq. (18), corresponds to hydrolysis starting, when there is only the polysaccharide within the molecular mass class *M*_0_^1^.

## Results and discussions

### Acid hydrolysis of cellulose consisting of 10 units of cellobiose

The stochastic model was applied to describe a hypothetical hydrolysis of a polysaccharide species (*s* = 1) consisting of 10 units of cellobiose. As shown in Fig. 2, 10 uniformly distributed molecular mass classes (*M*_0_^1^ = 3258 g/mol, *M*_1_^1^ = 2934 g/mol, *M*_2_^1^ = 2610 g/mol,…, *M*_8_^1^ = 666 g/mol, and *M*_9_^1^ = 342 g/mol) were taken into account. Moreover, cellobiose disaccharide (*M*_9_^1^ = 342 g/mol) was hydrolyzed resulting in 2 molecules of glucose (*M*_10_^1^ = 180 g/mol). Schema in Fig. 2 is based on data reported in the related literature^28,34^.

16 $$p^{1} \left( {\tau_{n} } \right) = [p_{0}^{{\begin{array}{*{20}c} {} \\ \end{array} 1}} \left( {\tau_{n} } \right) \, p_{1}^{{\begin{array}{*{20}c} {} \\ \end{array} 1}} \left( {\tau_{n} } \right) \, p_{2}^{{\begin{array}{*{20}c} {} \\ \end{array} 1}} \left( {\tau_{n} } \right) \ldots p_{8}^{{\begin{array}{*{20}c} {} \\ \end{array} 1}} \left( {\tau_{n} } \right) \, p_{9}^{{\begin{array}{*{20}c} {} \\ \end{array} 1}} \left( {\tau_{n} } \right) \, p_{10}^{{\begin{array}{*{20}c} {} \\ \end{array} 1}} \left( {\tau_{n} } \right)]$$

17 $$\tau_{n} = n\Delta t$$

18 $$p^{1} \left( {\tau_{0} } \right) = [p_{0}^{{\begin{array}{*{20}c} {} \\ \end{array} 1}} \left( {\tau_{0} } \right) \, p_{1}^{{\begin{array}{*{20}c} {} \\ \end{array} 1}} \left( {\tau_{0} } \right) \, p_{2}^{{\begin{array}{*{20}c} {} \\ \end{array} 1}} \left( {\tau_{0} } \right) \ldots p_{8}^{{\begin{array}{*{20}c} {} \\ \end{array} 1}} \left( {\tau_{0} } \right) \, p_{9}^{{\begin{array}{*{20}c} {} \\ \end{array} 1}} \left( {\tau_{0} } \right) \, p_{10}^{{\begin{array}{*{20}c} {} \\ \end{array} 1}} \left( {\tau_{0} } \right)] = [1 \, 0 \, 0 \ldots 0 \, 0 \, 0]$$

According to Markov stochastic cellular models, a transition probability matrix, *P*^1^, containing values of transition probability *P*_*ji*_^1^, is defined by Eq. (19) ^30^. *P*^1^ can be determined using different probability density functions of random variable. The breaking of a cellulose species within the molecular mass class *M*_0_^1^ can occur in the position *k* in the chain, where *k* = 1, 2…*K* − 1 and *K* is its polymerization degree (*PD*), resulting in two hydrolysates, one of them having *PD* = *k*, the other one *PD* = *K* − *k*. If *k* = 1, the breaking of the hydrolysate with *PD* = *K* − 1 can take place in the position *k*, where *k* = 1, 2…*K* − 2. Accordingly, the values of random variable are 1, 2…*K* − *i*, where *i* ≥ 1.

19 $$P^{1} = \left[ {\begin{array}{*{20}l} {P_{{00}}^{{\begin{array}{*{20}c} {} \\ \end{array} 1}} } \hfill & 0 \hfill & 0 \hfill & 0 \hfill & 0 \hfill & 0 \hfill & 0 \hfill & 0 \hfill & 0 \hfill & 0 \hfill & 0 \hfill \\ {P_{{10}}^{{\begin{array}{*{20}c} {} \\ \end{array} 1}} } \hfill & {P_{{11}}^{{\begin{array}{*{20}c} {} \\ \end{array} 1}} {\text{ }}} \hfill & 0 \hfill & 0 \hfill & 0 \hfill & 0 \hfill & 0 \hfill & 0 \hfill & 0 \hfill & 0 \hfill & 0 \hfill \\ {P_{{20}}^{{\begin{array}{*{20}c} {} \\ \end{array} 1}} } \hfill & {P_{{21}}^{{\begin{array}{*{20}c} {} \\ \end{array} 1}} } \hfill & {P_{{22}}^{{\begin{array}{*{20}c} {} \\ \end{array} 1}} } \hfill & 0 \hfill & 0 \hfill & 0 \hfill & 0 \hfill & 0 \hfill & 0 \hfill & 0 \hfill & 0 \hfill \\ {P_{{30}}^{{\begin{array}{*{20}c} {} \\ \end{array} 1}} } \hfill & {P_{{31}}^{{\begin{array}{*{20}c} {} \\ \end{array} 1}} } \hfill & {P_{{32}}^{{\begin{array}{*{20}c} {} \\ \end{array} 1}} } \hfill & {P_{{33}}^{{\begin{array}{*{20}c} {} \\ \end{array} 1}} } \hfill & 0 \hfill & 0 \hfill & 0 \hfill & 0 \hfill & 0 \hfill & 0 \hfill & 0 \hfill \\ {P_{{40}}^{{\begin{array}{*{20}c} {} \\ \end{array} 1}} } \hfill & {P_{{41}}^{{\begin{array}{*{20}c} {} \\ \end{array} 1}} } \hfill & {P_{{42}}^{{\begin{array}{*{20}c} {} \\ \end{array} 1}} } \hfill & {P_{{43}}^{{\begin{array}{*{20}c} {} \\ \end{array} 1}} } \hfill & {P_{{44}}^{{\begin{array}{*{20}c} {} \\ \end{array} 1}} } \hfill & 0 \hfill & 0 \hfill & 0 \hfill & 0 \hfill & 0 \hfill & 0 \hfill \\ {P_{{50}}^{{\begin{array}{*{20}c} {} \\ \end{array} 1}} } \hfill & {P_{{51}}^{{\begin{array}{*{20}c} {} \\ \end{array} 1}} } \hfill & {P_{{52}}^{{\begin{array}{*{20}c} {} \\ \end{array} 1}} } \hfill & {P_{{53}}^{{\begin{array}{*{20}c} {} \\ \end{array} 1}} } \hfill & {P_{{54}}^{{\begin{array}{*{20}c} {} \\ \end{array} 1}} } \hfill & {P_{{55}}^{{\begin{array}{*{20}c} {} \\ \end{array} 1}} } \hfill & 0 \hfill & 0 \hfill & 0 \hfill & 0 \hfill & 0 \hfill \\ {P_{{60}}^{{\begin{array}{*{20}c} {} \\ \end{array} 1}} } \hfill & {P_{{61}}^{{\begin{array}{*{20}c} {} \\ \end{array} 1}} } \hfill & {P_{{62}}^{{\begin{array}{*{20}c} {} \\ \end{array} 1}} } \hfill & {P_{{63}}^{{\begin{array}{*{20}c} {} \\ \end{array} 1}} } \hfill & {P_{{64}}^{{\begin{array}{*{20}c} {} \\ \end{array} 1}} } \hfill & {P_{{65}}^{{\begin{array}{*{20}c} {} \\ \end{array} 1}} {\text{ }}} \hfill & {P_{{66}}^{{\begin{array}{*{20}c} {} \\ \end{array} 1}} } \hfill & 0 \hfill & 0 \hfill & 0 \hfill & 0 \hfill \\ {P_{{70}}^{{\begin{array}{*{20}c} {} \\ \end{array} 1}} } \hfill & {P_{{71}}^{{\begin{array}{*{20}c} {} \\ \end{array} 1}} } \hfill & {P_{{72}}^{{\begin{array}{*{20}c} {} \\ \end{array} 1}} } \hfill & {P_{{73}}^{{\begin{array}{*{20}c} {} \\ \end{array} 1}} } \hfill & {P_{{74}}^{{\begin{array}{*{20}c} {} \\ \end{array} 1}} } \hfill & {P_{{75}}^{{\begin{array}{*{20}c} {} \\ \end{array} 1}} } \hfill & {P_{{76}}^{{\begin{array}{*{20}c} {} \\ \end{array} 1}} } \hfill & {P_{{77}}^{{\begin{array}{*{20}c} {} \\ \end{array} 1}} } \hfill & 0 \hfill & 0 \hfill & 0 \hfill \\ {P_{{80}}^{{\begin{array}{*{20}c} {} \\ \end{array} 1}} } \hfill & {P_{{81}}^{{\begin{array}{*{20}c} {} \\ \end{array} 1}} } \hfill & {P_{{82}}^{{\begin{array}{*{20}c} {} \\ \end{array} 1}} } \hfill & {P_{{83}}^{{\begin{array}{*{20}c} {} \\ \end{array} 1}} {\text{ }}} \hfill & {P_{{84}}^{{\begin{array}{*{20}c} {} \\ \end{array} 1}} } \hfill & {P_{{85}}^{{\begin{array}{*{20}c} {} \\ \end{array} 1}} } \hfill & {P_{{86}}^{{\begin{array}{*{20}c} {} \\ \end{array} 1}} } \hfill & {P_{{87}}^{{\begin{array}{*{20}c} {} \\ \end{array} 1}} } \hfill & {{\text{ }}P_{{88}}^{{\begin{array}{*{20}c} {} \\ \end{array} 1}} } \hfill & 0 \hfill & 0 \hfill \\ {P_{{90}}^{{\begin{array}{*{20}c} {} \\ \end{array} 1}} } \hfill & {P_{{91}}^{{\begin{array}{*{20}c} {} \\ \end{array} 1}} } \hfill & {P_{{92}}^{{\begin{array}{*{20}c} {} \\ \end{array} 1}} } \hfill & {P_{{93}}^{{\begin{array}{*{20}c} {} \\ \end{array} 1}} } \hfill & {P_{{94}}^{{\begin{array}{*{20}c} {} \\ \end{array} 1}} } \hfill & {P_{{95}}^{{\begin{array}{*{20}c} {} \\ \end{array} 1}} } \hfill & {P_{{96}}^{{\begin{array}{*{20}c} {} \\ \end{array} 1}} } \hfill & {P_{{97}}^{{\begin{array}{*{20}c} {} \\ \end{array} 1}} } \hfill & {P_{{98}}^{{\begin{array}{*{20}c} {} \\ \end{array} 1}} } \hfill & {P_{{99}}^{{\begin{array}{*{20}c} {} \\ \end{array} 1}} } \hfill & 0 \hfill \\ {P_{{100}}^{{\begin{array}{*{20}c} {} \\ \end{array} 1}} } \hfill & {P_{{101}}^{{\begin{array}{*{20}c} {} \\ \end{array} 1}} {\text{ }}} \hfill & {P_{{102}}^{{\begin{array}{*{20}c} {} \\ \end{array} 1}} } \hfill & {P_{{103}}^{{\begin{array}{*{20}c} {} \\ \end{array} 1}} } \hfill & {P_{{104}}^{{\begin{array}{*{20}c} {} \\ \end{array} 1}} } \hfill & {P_{{105}}^{{\begin{array}{*{20}c} {} \\ \end{array} 1}} } \hfill & {P_{{106}}^{{\begin{array}{*{20}c} {} \\ \end{array} 1}} } \hfill & {P_{{107}}^{{\begin{array}{*{20}c} {} \\ \end{array} 1}} } \hfill & {P_{{108}}^{{\begin{array}{*{20}c} {} \\ \end{array} 1}} } \hfill & {P_{{109}}^{{\begin{array}{*{20}c} {} \\ \end{array} 1}} } \hfill & 1 \hfill \\ \end{array} } \right]$$

Assuming the highest breaking probability in the centre of polymeric chain, a Gauss (normal) distribution can be used to compute the probabilities in the transition probability matrix. A random variable *X* that takes values *x* is normally (Gauss) distributed when the probability density function is expressed by Eq. (20), where *μ* is the mean of *x* values, *σ*^2^ the variance, and *σ* the standard deviation.

20 $$f_{GS} (x) = \frac{1}{{\sigma \sqrt {2\pi } }}\exp \left( { - \frac{{(x - \mu )^{2} }}{{2\sigma^{2} }}} \right)$$

Normal distributions have many useful properties, so random variables with unknown distributions are often considered to be normal, especially in chemistry, physics, biology, etc. This assumption is based on the central limit theorem (CLT), which states that if sufficiently large random samples are taken from a population having any distribution of a variable and finite values of mean and variance, then the distribution of sample mean will approach a Gauss (normal) distribution. There is an obvious tendency in sciences and social live to assume normal distributions in applications where they may not be suitable. As Lippmann concluded, “Everybody believes in the exponential law of errors: the experimenters, because they think it can be proved by mathematics; and the mathematicians, because they believe it has been established by observation”^35^.

**Figure 3:**
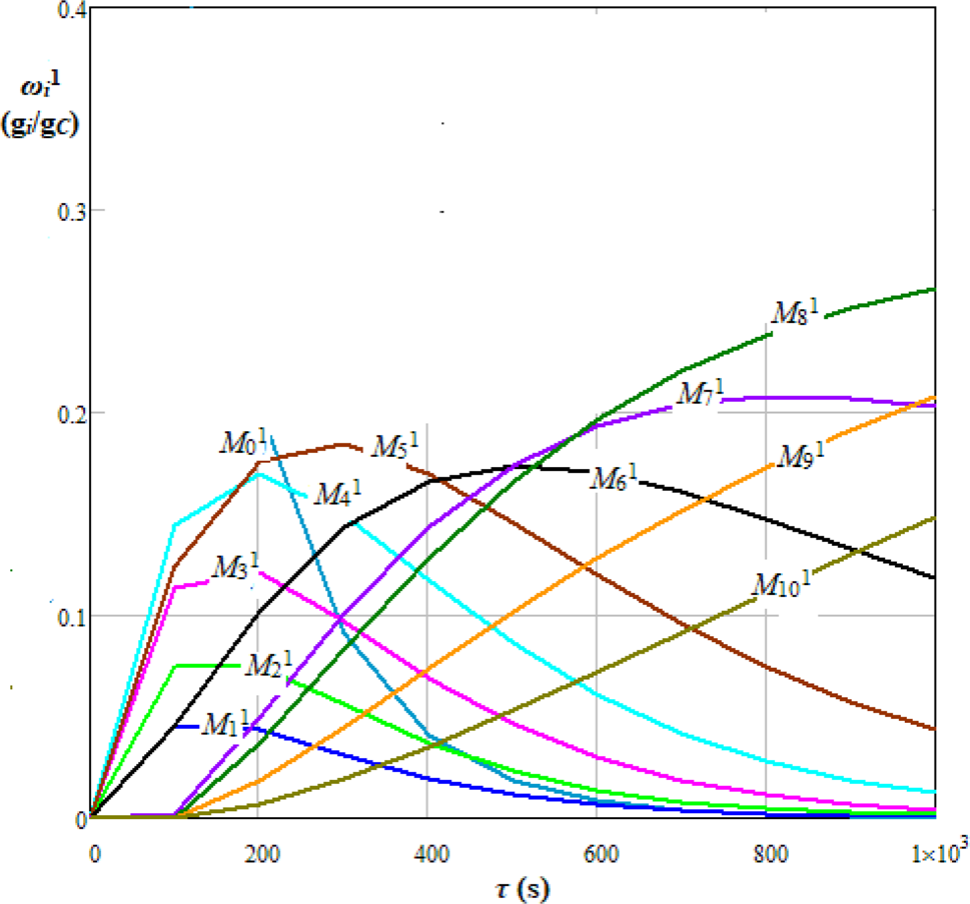
Dynamics of mass fraction of *i* hydrolysable species within the molecular mass class *M*_*i*_^1^ (*i* = 0, 1…10), *ω*_*i*_^1^, for Gauss (normal) distribution of transition probabilities.

Considering the mean (*μ*) and standard deviation (*σ*) given by Eqs. (21) and (22), the elements of transition probability matrix, *P*_*ji*_^1^, based on a Gauss distribution can be determined by Eq. (23). For the hydrolysis case depicted in Fig. 2 (*i* = 0, 1…*N*, *N* = 10), *P*^1^ is given by Eq. (24). Dynamics of mass fraction of *i* hydrolysable species within the molecular mass class *M*_*i*_^1^ (*i* = 0, 1…10), *ω*_*i*_^1^(*τ*), which were obtained based on Eqs. (13)−(15) and (24), where Δ*τ* = 100 s, are shown in Fig. 3.

21 $$\mu = \mu (x) = \frac{(N + 1 - x)}{2}$$

22 $$\sigma = \sigma (x) = \sqrt {\frac{{(N + 1 - x)^{2} - 1}}{12}}$$

23 $$P_{ji}^{{\begin{array}{*{20}c} {} \\ \end{array} 1}} = \left\{ {\begin{array}{*{20}l} {\frac{1}{N + 1}} \hfill & {{\text{if }}i = j = {0}} \hfill \\ {1 } \hfill & {{\text{ if }}i = j = N} \hfill \\ {pp_{ji}^{{\begin{array}{*{20}c} {} \\ \end{array} 1}} = \left| {\begin{array}{*{20}l} {f_{GS} \left( {j - i} \right) = \frac{1}{{\sigma \sqrt {2\pi } }}\exp \left( { - \frac{{(j - i - \mu )^{2} }}{{2\sigma^{2} }}} \right)} \hfill & {{\text{ if }}i < j} \hfill \\ 0 \hfill & {\text{ otherwise}} \hfill \\ \end{array} } \right.} \hfill & {{\text{if }}i \ne j \, } \hfill \\ {{1} - \sum\limits_{{j = {1}}}^{N} {pp_{ji}^{{\begin{array}{*{20}c} {} \\ \end{array} 1}} } } \hfill & {\text{ otherwise}} \hfill \\ \end{array} } \right.$$

24 $$P^{1} = \left[ {\begin{array}{*{20}l} {0.{318}} \hfill & {0} \hfill & {0} \hfill & {0} \hfill & {0} \hfill & {0} \hfill & {0} \hfill & {0} \hfill & {0} \hfill & {0} \hfill & {0} \hfill \\ {0.036} \hfill & {0.352} \hfill & {0} \hfill & {0} \hfill & {0} \hfill & {0} \hfill & {0} \hfill & {0} \hfill & {0} \hfill & {0} \hfill & {0} \hfill \\ {0.066} \hfill & {0.036} \hfill & {0.352} \hfill & {0} \hfill & {0} \hfill & {0} \hfill & {0} \hfill & {0} \hfill & {0} \hfill & {0} \hfill & {0} \hfill \\ {{0}{\text{.114 }}} \hfill & {{0}{\text{.066}}} \hfill & {{0}{\text{.036 }}} \hfill & {{0}{\text{.352 }}} \hfill & {0} \hfill & {0} \hfill & {0} \hfill & {0} \hfill & {0} \hfill & {0} \hfill & {0} \hfill \\ {0.170} \hfill & {0.114 \, } \hfill & { \, 0.066} \hfill & {0.036} \hfill & {0.357 \, } \hfill & {0} \hfill & {0} \hfill & {0} \hfill & {0} \hfill & {0} \hfill & {0} \hfill \\ {{0}{\text{.176}}} \hfill & {{0}{\text{.170 }}} \hfill & {{0}{\text{.114}}} \hfill & {{ 0}{\text{.066}}} \hfill & {{0}{\text{.036 }}} \hfill & {{0}{\text{.437 }}} \hfill & {0} \hfill & {0} \hfill & {0} \hfill & {0} \hfill & {0} \hfill \\ {{0}{\text{.080 }}} \hfill & {{0}{\text{.176 }}} \hfill & {{0}{\text{.170}}} \hfill & {{0}{\text{.114}}} \hfill & {{0}{\text{.066 }}} \hfill & {{ 0}{\text{.036}}} \hfill & {{0}{\text{.613 }}} \hfill & {0} \hfill & {0} \hfill & {0} \hfill & {0} \hfill \\ {0.005} \hfill & {{0}{\text{.080}}} \hfill & {{0}{\text{.176}}} \hfill & {{0}{\text{.170}}} \hfill & {{ 0}{\text{.114}}} \hfill & {{0}{\text{.066 }}} \hfill & {{ 0}{\text{.036}}} \hfill & {{0}{\text{.783}}} \hfill & {0} \hfill & {0} \hfill & {0} \hfill \\ {0.000} \hfill & {0.005 \, } \hfill & {{ 0}{\text{.080}}} \hfill & {{0}{\text{.176}}} \hfill & {{0}{\text{.170}}} \hfill & {{0}{\text{.114}}} \hfill & {{0}{\text{.066}}} \hfill & {{ 0}{\text{.036 }}} \hfill & {{0}{\text{.898}}} \hfill & {0} \hfill & {0} \hfill \\ {0.000} \hfill & {{0}{\text{.000}}} \hfill & {0.005} \hfill & {{0}{\text{.080}}} \hfill & {{0}{\text{.176 }}} \hfill & {{0}{\text{.170 }}} \hfill & {{ 0}{\text{.114 }}} \hfill & {{0}{\text{.066}}} \hfill & {{ 0}{\text{.036}}} \hfill & {{0}{\text{.964}}} \hfill & {0} \hfill \\ {{0}{\text{.000 }}} \hfill & {0.000} \hfill & {{0}{\text{.000}}} \hfill & {0.005 \, } \hfill & {{0}{\text{.080}}} \hfill & {{ 0}{\text{.176}}} \hfill & {{ 0}{\text{.170}}} \hfill & {{0}{\text{.114 }}} \hfill & {{0}{\text{.066}}} \hfill & {{ 0}{\text{.036}}} \hfill & {1 } \hfill \\ \end{array} } \right]$$

Many dynamic phenomena in the chemical engineering field have been characterized and modelled using Weibull distribution^36–38^. Probability density function of a Weibull random variable is given by Eq. (25), where *β* > 0 is the shape parameter, *λ* > 0 is the scale parameter, and *x* ≥ 0 the values of Weibull random variable^39^.

25 $$f_{W} (x) = \frac{\beta }{\lambda }\left( {\frac{x}{\lambda }} \right)^{\beta - 1} \exp \left( { - \left( {\frac{x}{\lambda }} \right)^{\beta } } \right)$$

**Figure 4:**
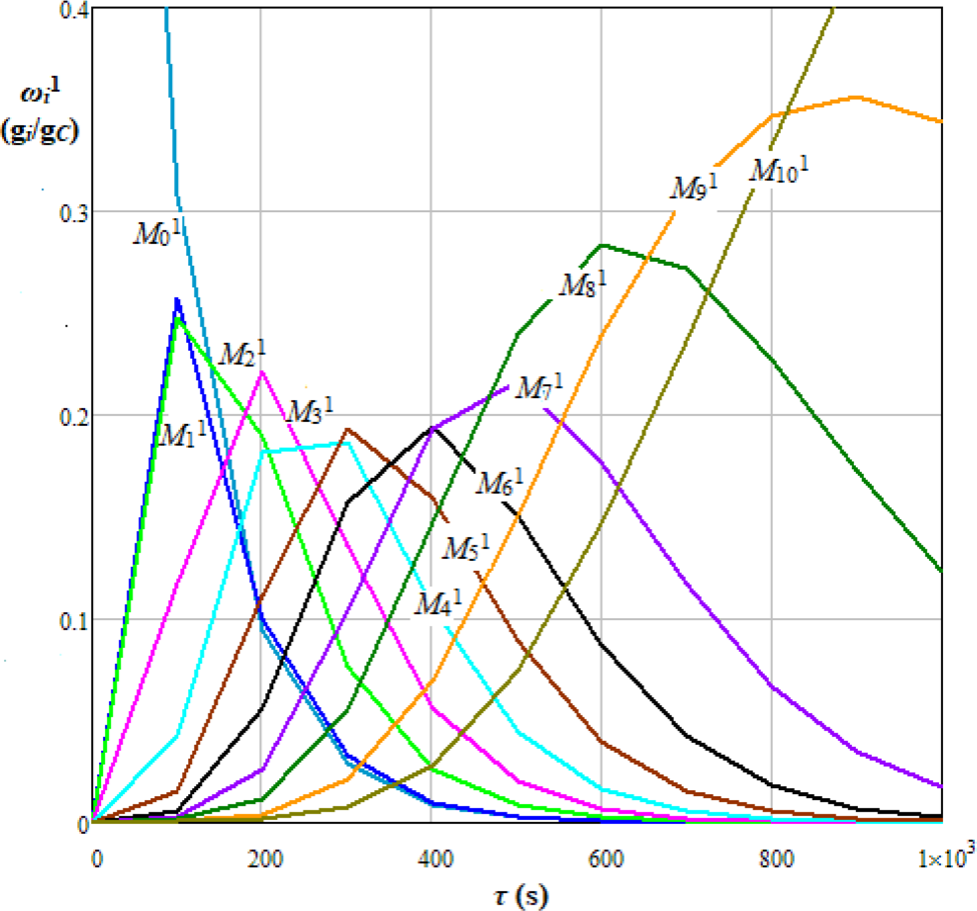
Dynamics of mass fraction of *i* hydrolysable species within the molecular mass class *M*_*i*_^1^ (*i* = 0, 1…10), *ω*_*i*_^1^, for Weibull distribution of transition probabilities.

In the case of cellulose hydrolysis, the values of shape parameter are interpreted as follows: (i) 0 < *β* < 1 indicates that the chain breaking rate decreases over time, meaning that the process hydrolysates become more stable, (ii) *β* = 1 implies a constant breaking rate over time, and (iii) *β* > 1 corresponds to an increase in the breaking rate. Characteristic parameters of Weibull distribution were expressed by Eqs. (26) and (27) depending on the integer values of Weibull random variable (*x* ≥ 0), *i.e.*, scale parameter (*λ*) was linked to the mean (*μ*) of random variable values and shape parameter (*β* > 0.5) to the variance (*σ*^2^). For the hydrolysis case presented in Fig. 2, the elements of transition probability matrix, *P*_*ji*_^1^, are given by Eq. (28). Dynamics of mass fraction of *i* hydrolysable species within the molecular mass class *M*_i_^1^ (*i* = 0, 1…10), *ω*_*i*_^1^(*τ*), obtained based on Eqs. (13)−(15), (26)−(28), where Δ*τ* = 100 s, are shown in Fig. 4.

26 $$\lambda = \lambda (x) = 1.4 + \frac{\mu (x)}{N}$$

27 $$\beta = \beta (x) = 0.95 + \frac{{\sigma^{2} (x)}}{N}$$

28 $$P_{ji}^{{\begin{array}{*{20}c} {} \\ \end{array} 1}} = \left\{ {\begin{array}{*{20}l} {\frac{3.5}{{N + 1}}} \hfill & {{\text{if }}i = j = {0}} \hfill \\ {1 } \hfill & {{\text{if }}i = j = N} \hfill \\ {pp_{ji}^{{\begin{array}{*{20}c} {} \\ \end{array} 1}} = \left\{ {\begin{array}{*{20}l} {f_{W} \left( {j - i} \right) = \frac{\beta }{\lambda }\left( {\frac{j - i}{\lambda }} \right)^{\beta - 1} \exp \left( { - \left( {\frac{j - i}{\lambda }} \right)^{\beta } } \right)} \hfill & {{\text{if }}i < j} \hfill \\ {0} \hfill & {{\text{otherwise}}} \hfill \\ \end{array} } \right.} \hfill & {{\text{ if }}i \ne j \, } \hfill \\ {{1} - \sum\limits_{{j = {1}}}^{N} {pp_{ji}^{{\begin{array}{*{20}c} {} \\ \end{array} 1}} \, } } \hfill & {{\text{otherwise}}} \hfill \\ \end{array} } \right.$$

### Acid hydrolysis of cellulose consisting of 100 units of cellobiose

**Figure 5:**
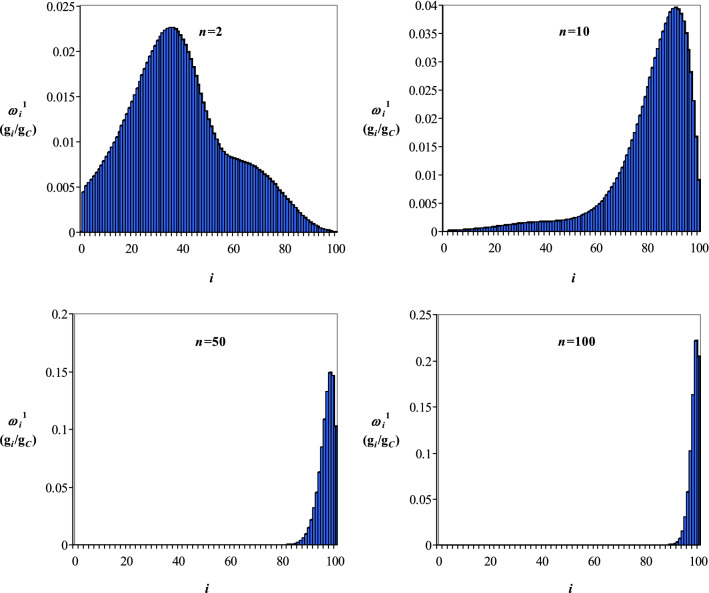
Mass fractions of *i* hydrolysable species within the molecular mass class *M*_*i*_^1^ (*i* = 0, 1…100), *ω*_*i*_^1^, at different time sequences (*n* = 2, 10, 50, 100) for hydrolysis of a cellulose with *PD* = 200 (100 units of CB) assuming a Gauss distribution of transition probabilities.

**Figure 6:**
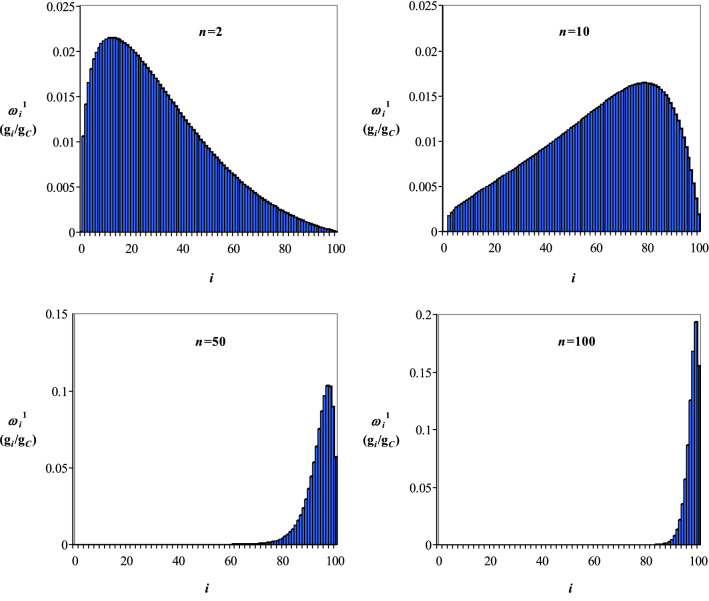
Mass fractions of *i* hydrolysable species within the molecular mass class *M*_*i*_^1^ (*i* = 0, 1…100), *ω*_*i*_^1^, at different time sequences (*n* = 2, 10, 50, 100) for hydrolysis of a cellulose with *PD* = 200 (100 units of CB) assuming a Weibull distribution of transition probabilities.

Mass fractions of *i* hydrolysable species, *ω*_*i*_^1^ (g_*i*_/g_*C*_), at different time sequences for acid hydrolysis of cellulose with a value of *PD* of 200 (corresponding to 100 units of CB), are shown in Figs. 5 and 6, where Gauss (Fig. 5) and Weibull (Fig. 6) distributions of transition probabilities were considered. The results presented in Figs. 5 and 6 highlight a relatively quick disappearance of macromolecules with high molecular mass, suggesting that the hydrolysis of species with low molecular mass is the rate-limiting step. This finding is in line with the conclusions of other researches, where the cellulose hydrolysis was assumed as a homogeneous kinetic process, occurred according to scheme (29) and described by Eqs. (30)−(32), where *c*_*C*_, *c*_*G*_, and *c*_*HMF*_ are the concentrations of cellulose (C), glucose (G), and hydroxymethylfurfural (HMF), *k*_1_ and *k*_2_ represent the rate constants, and *τ* is the time^40–43^.

29 $${\text{Cellulose }}\left( {\text{C}} \right)\mathop{\longrightarrow}\limits^{{k_{1} }}{\text{Glucose }}\left( {\text{G}} \right)\mathop{\longrightarrow}\limits^{{k_{2} }}{\text{Hydroxymethylfurfural }}\left( {{\text{HMF}}} \right)$$

30 $$\frac{{dc_{C} }}{d\tau } = - k_{1} c_{C}$$

31 $$\frac{{dc_{G} }}{d\tau } = k_{1} c_{C} - k_{2} c_{G}$$

32 $$\frac{{dc_{HMF} }}{d\tau } = k_{2} c_{G}$$

Acid hydrolysis dynamics are heavily affected by process temperature, acid type and concentration. Arrhenius equations [Eqs. (33) and (34)], where the contribution of acid concentration (divided by a reference value, *c*_*a*_/*c*_*a,ref*_) was taken into account, were used to express characteristic reaction rate constants of homogeneous kinetic model (*k*_1_ and *k*_2_) depending on absolute temperature (*T*).

33 $$k_{1} = k_{10} \left( {\frac{{c_{a} }}{{c_{a,ref} }}} \right)^{{n_{1} }} \exp \left( { - \frac{{E_{A1} }}{RT}} \right)$$

34 $$k_{2} = k_{20} \left( {\frac{{c_{a} }}{{c_{a,ref} }}} \right)^{{n_{2} }} \exp \left( { - \frac{{E_{A2} }}{RT}} \right)$$

According to Eqs. (33) and (34), transition probability frequencies in the stochastic model, *α*_*ji*_^1^, can be estimated by Eq. (35), where *E*_*Am*_ represents a mean reaction activation energy (taking into account all decompositions to glucose), *E*_*AG*_ is the activation energy for transition from glucose (G) to HMF, and *T*_*ref*_ is a reference absolute temperature.

35 $$\alpha_{ji}^{{\begin{array}{*{20}c} {} \\ \end{array} 1}} = \left\{ \begin{gathered} \alpha_{ji0}^{{\begin{array}{*{20}c} {} \\ \end{array} \begin{array}{*{20}c} {} \\ \end{array} 1}} \left( {\frac{{c_{a} }}{{c_{a,ref} }}} \right)^{n} \exp \left[ {\frac{{E_{Am} }}{R}\left( {\frac{1}{T} - \frac{1}{{T_{ref} }}} \right)} \right]{, }i{,}j = {0,1}...N - 1 \hfill \\ \alpha_{ji0}^{{\begin{array}{*{20}c} {} \\ \end{array} \begin{array}{*{20}c} {} \\ \end{array} 1}} \left( {\frac{{c_{a} }}{{c_{a,ref} }}} \right)^{n} \exp \left[ {\frac{{E_{AG} }}{R}\left( {\frac{1}{T} - \frac{1}{{T_{ref} }}} \right)} \right]{, }i = N - 1{,}j = N \hfill \\ \end{gathered} \right.$$

**Figure 7:**
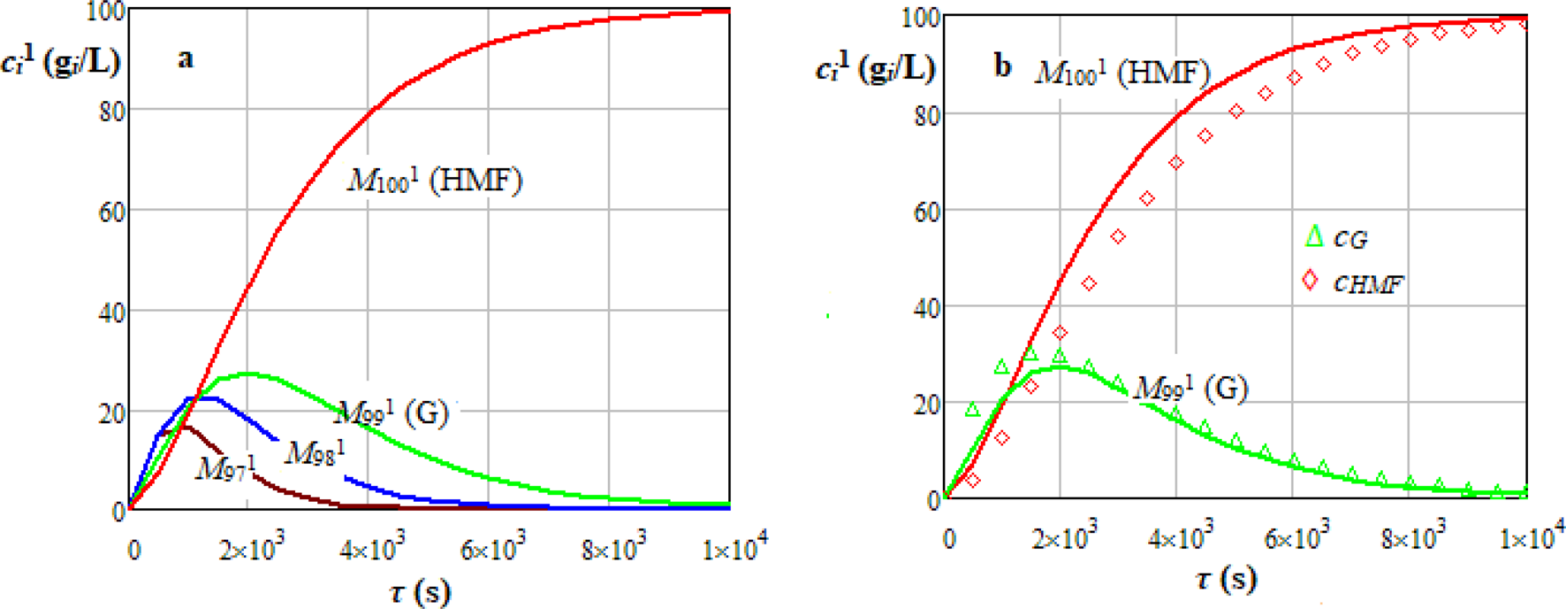
Dynamics of *i* hydrolysable species (*M*_97_^1^…*M*_100_^1^) concentration, *c*_*i*_^1^ (g_*i*_/L), predicted by stochastic model for sulphuric acid hydrolysis of a cellulose (C) suspension (*PD* = 200, *c*_*C*0_ = 100 g_*C*_/L, ∆*τ* = 10 s, Gauss distributed transition probabilities) (**a**) and comparison between stochastic model (line) and homogeneous deterministic kinetic model (points) for *M*_99_^1^ and *M*_100_^1^ (*T* = 483 K, *T*_*ref*_ = 453 K, *c*_*a*_ = 4%, *c*_*a,ref*_ = 1%) (**b**).

**Figure 8:**
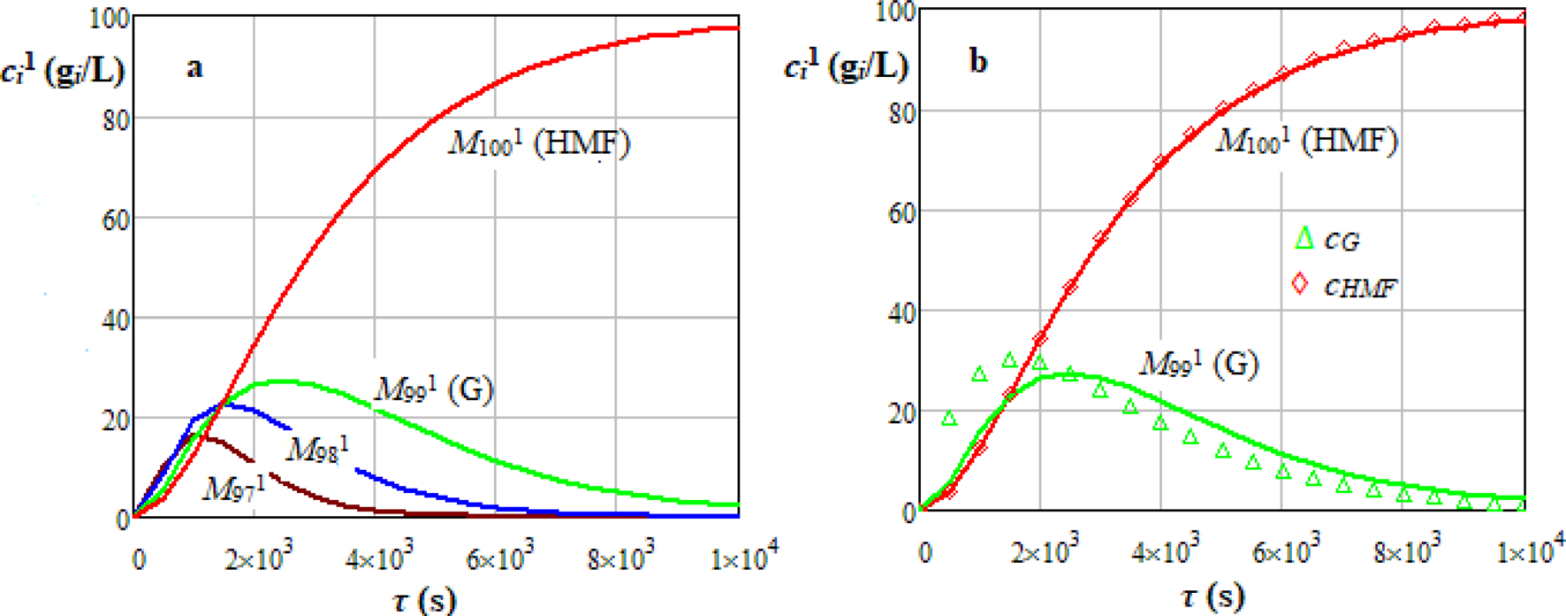
Dynamics of *i* hydrolysable species (*M*_97_^1^…*M*_100_^1^) concentration, *c*_*i*_^1^ (g_i_/L), predicted by stochastic model for sulphuric acid hydrolysis of a cellulose (C) suspension (*PD* = 200, *c*_*C*0_ = 100 g_*C*_/L, ∆*τ* = 10 s, Weibull distributed transition probabilities) (**a**) and comparison between stochastic model (line) and homogeneous deterministic kinetic model (points) for *M*_99_^1^ and *M*_100_^1^ (*T* = 483 K, *T*_*ref*_ = 453 K, *c* = 4%, *c*_*a,ref*_ = 1%) (**b**).

Dynamics of *i* hydrolysable species concentration, *c*_*i*_^1^ (g_*i*_/L), predicted by stochastic model for cellulose hydrolysis are shown in Figs. 7a and 8a, where the matrix of transition probabilities, based on Gauss or Weibull distributions, was determined according to Eqs. (36)−(39). Dynamics of *c*_*G*_ and *c*_*HMF*_ predicted by a homogeneous deterministic kinetic model [Eqs. (30)−(34)] based on data reported in the related literature^40–43^ are represented in Figs. 7b and 8b. Values of characteristic parameters of deterministic and stochastic models are specified in Table S1^5^. The largest values of root mean square error (*RMSE*) and coefficient of variation (*CV*) for results presented in Figs. 7b and 8b are summarized in Table S2. Data predicted by deterministic and stochastic models for a cellulose with *PD* = 200 assuming Gauss and Weibull distributed transition probabilities were in a reasonable agreement, i.e., *RMSE* ≤ 6.50 g_*i*_/L (*CV* ≤ 0.098) and *RMSE* ≤ 5.26 g_*i*_/L (*CV* ≤ 0.410).

36 $$P_{ji}^{{\begin{array}{*{20}c} {} \\ \end{array} 1}} = \left\{ \begin{gathered} \frac{1}{N + 1}{\text{ if }}i = j = {0} \hfill \\ {\text{1 if }}i = j = N \hfill \\ pp_{ji}^{{\begin{array}{*{20}c} {} \\ \end{array} 1}} = \left\{ \begin{gathered} f_{GS} \left( {j - i} \right)g\left( {c_{a} } \right)h\left( T \right){\text{ if }}i < j \hfill \\ {\text{0 otherwise}} \hfill \\ \end{gathered} \right.{\text{ if }}i \ne j \, \hfill \\ {1} - \sum\limits_{{j = {1}}}^{N} {pp_{ji}^{{\begin{array}{*{20}c} {} \\ \end{array} 1}} {\text{ otherwise }}} \hfill \\ \end{gathered} \right.$$

37 $$P_{ji}^{{\begin{array}{*{20}c} {} \\ \end{array} 1}} = \left\{ {\begin{array}{*{20}l} {\frac{3.5}{{N + 1}}} \hfill & {{\text{if }}i = j = {0}} \hfill \\ {1 } \hfill & {{\text{if }}i = j = N} \hfill \\ {pp_{ji}^{1} = \left\{ {\begin{array}{*{20}c} {f_{W} \left( {j - i} \right)g\left( {c_{a} } \right)h\left( T \right)} & {{\text{ if }}i < j} \\ {0} & {{\text{otherwise}}} \\ \end{array} } \right.} \hfill & {{\text{ if }}i \ne j \, } \hfill \\ {{1} - \sum\limits_{{j = {1}}}^{N} {pp_{ji}^{1} } } \hfill & {\text{ otherwise}} \hfill \\ \end{array} } \right.$$

38 $$g\left( {c_{a} } \right) = \left( {\frac{{c_{a} }}{{c_{a,ref} }}} \right)^{n}$$

39 $$h\left( T \right) = \left\{ {\begin{array}{*{20}l} {\exp \left[ {\frac{{E_{Am} }}{R}\left( {\frac{1}{T} - \frac{1}{{T_{ref} }}} \right)} \right]{,}} \hfill & { \, i{,}j = {0,1}...N - 1} \hfill \\ {\exp \left[ {\frac{{E_{AG} }}{R}\left( {\frac{1}{T} - \frac{1}{{T_{ref} }}} \right)} \right]{,}} \hfill & {i = N - 1{,}j = N} \hfill \\ \end{array} } \right.$$

## Conclusions

The production of cellulosic biofuel, which is technologically feasible, expects the transition to industrial scale. Accordingly, the hydrolysis of cellulose and (ligno)cellulosic biomass has been of great interest lately. Numerous experimental and theoretical studies on acid hydrolysis have been reported in the related literature. Deterministic kinetic models are commonly applied to describe the acid hydrolysis.

Two stochastic models, one involving a Gauss distribution of transition probabilities, the other a Weibull distribution, were developed in this paper to predict the kinetics of acid hydrolysis of cellulose. It was assumed that the cellulose subjected to the hydrolysis contained a finite number of hydrolysable polysaccharide species (cellulose fragments with different *PD*), random breaking of a polysaccharide was based on the cleavage of a parent macromolecule into two descendants, and the molecular fragmentation was a homogeneous Markov process. Model equations, transition probability matrix and dynamics of mass fraction of hydrolysable species for a cellulose consisting of 10 units of CB (*PD* = 20) were presented in Methods and the first part of Results and discussions. The models were then extended, considering the effects of acid concentration and process temperature on the kinetics of acid hydrolysis of a cellulose consisting of 100 units of CB (*PD* = 200). Dynamics of mass concentration of hydrolysable species predicted by stochastic models and those determined using a homogeneous deterministic kinetic model were compared and a reasonable agreement was obtained. Both stochastic models can accurately predict the kinetics of acid hydrolysis and cover the limitations of some deterministic kinetic models, *e.g*., large number of equations and parameters, modification of parameter values by changing the process conditions.

## Supplementary Information

Supplementary Information.

## Nomenclature

*c*_*a*_: Acid concentration (%)
*c*_*C*_: Concentration of cellulose in the reaction medium (g_*C*_/L)
*c*_*CB*_: Concentration of cellobiose in the reaction medium (g_*CB*_/L)
*c*_*C*__0_: Initial concentration of cellulose in the reaction medium (g_*C*_/L)
*c*_*E*__1_: Concentration of enzymes *E*1 in the reaction medium (FPU/L) (FPU-filter paper units)
*c*_*G*_: Concentration of glucose in the reaction medium (g_*G*_/L)
*c*_*HMF*_: Concentration of hydroxymethylfurfural in the reaction medium (g_*HMF*_/L)
*c*_*i*_^*s*^: Concentration of *i* hydrolysable species within the molecular mass class *M*_*i*_^*s*^ (g_*i*_/L)
*CV*: Coefficient of variation
*E*_*A*_: Activation energy (J/mol)
*f*: Probability density function
*k*_*j*_: Kinetic constant for *j* reaction (s^−^^1^)
*K*_*L*_: Langmuir constant for sorption equilibrium (FPU/L)
*k*_max__*j*_: Maximum value of *k*_*j*_ at full saturation of the substrate with enzymes *E*1 (s^−^^1^)
*M*_*i*_^*s*^: Molecular mass class (range) of *s* polysaccharide species (g_*i*_/mol)
*PD*: Polymerization degree
*P*_*ji*_^*s*^: Transition probability of *s* polysaccharide species from *M*_*i*_^*s*^ to *M*_*j*_^*s*^
*p*^*s*^: Probability of breaking of *s* polysaccharide species
*P*^*s*^: Transition probability matrix of *s* polysaccharide species
*R*: Ideal gas constant (8.314 J/mol/K)
*RMSE*: Root mean square error (g_*i*_/L)
*T*: Absolute temperature (*K*)
*v*_*R*_: Reaction rate (g/L/s)
*X*: Random variable
*x*: Value of *X*
*α*_*ji*_^*s*^: Transition probability frequency of *s* polysaccharide species (s^-1^)
*β*: Shape parameter
*λ*: Scale parameter
*μ*: Mean of *x* values
*σ*: Standard deviation
*σ*^2^: Variance
*τ*: Time (s)
*ω*_*i*_^*s*^: Mass fraction of *s* polysaccharide species within *M*_*i*_^*s*^ (g_*i*_/g_*C*_)

## Subscripts

*C*: Cellulose
*CB*: Cellobiose
*G*: Glucose
*GS*: Gauss
*HMF*: Hydroxymethylfurfural
*ref*: Reference
*W*: Weibull

## Superscript

*s*: Polysaccharide species (*s* = 1, 2*…S*)
