# Supplementary material for: Stochastic modelling of cellulose hydrolysis with Gauss and Weibull distributed transition probabilities
Source: Sci Rep. 2021 May 4;11:9466. doi: 10.1038/s41598-021-88873-6 (PMC8097066; doi:10.1038/s41598-021-88873-6)
Supplement: Supplementary file 1 — Supplementary Information. [file 41598_2021_88873_MOESM1_ESM.doc]

**Stochastic modelling of cellulose hydrolysis with Gauss and Weibull distributed transition probabilities**

**Joseph Mcgreg Duru, Oana Cristina Pârvulescu^*^, Tănase Dobre, Cristian Eugen Răducanu**

University POLITEHNICA of Bucharest, Chemical and Biochemical Engineering Department, 1-6 Gheorghe Polizu, 011061, Bucharest, Romania

**^*^**Corresponding author. E-mail address: oana.parvulescu@yahoo.com.

**Supplementary tables**

**Table S1.** Values of parameters in Eqs. (33)−(35)

| Model | Parameter | Symbol | Value |
| --- | --- | --- | --- |
| Stochastic | Mean activation energy of breaking process in Eq. (35) | *E_Am_* | 26.6 kJ/mol |
|  | Activation energy for transition from glucose to HMF in Eq. (35) | *E_AG_* | 31.2 kJ/mol |
|  | Reference temperature | *T_ref_* | 453 K |
|  | Reference acid concentration | *c_a,ref_* | 1% (w/w) |
|  | Power in Eq. (35) | *n* | 0.3 |
| Deterministic | Constant in Eq. (33) | *k*_10_ | 4.93×10^-4^ s^-1^ |
|  | Constant in Eq. (34) | *k*_20_ | 6.41×10^-4^ s^-1^ |
|  | Activation energy in Eq. (33) | *E_A_*_1_ | 66.96 J/mol |
|  | Activation energy in Eq. (34) | *E_A_*_2_ | 82.41 J/mol |
|  | Power in Eq. (33) | *n*_1_ | 0.4 |
|  | Power in Eq. (34) | *n*_2_ | 0.5 |

**Table S2.** Comparison between data predicted by deterministic model (DM) and stochastic model (SM)

| No. | *τ* (s) | Concentration of HMF  (g*_i_*/L) in Fig. 7b | | Concentration of glucose (g*_i_*/L) in Fig. 8b | |
| --- | --- | --- | --- | --- | --- |
|  |  | DM | SM (Gauss distribution) | DM | SM (Weibull distribution) |
|  |  | *c_HMF,DM_* | *c_HMF,GS_* | *c_G,DM_* | *c_G,W_* |
| 1 | 0 | 0 | 0 | 0 | 0 |
| 2 | 500 | 2.5 | 4.1 | 18.9 | 4.1 |
| 3 | 1000 | 11.1 | 20.0 | 28.1 | 14.4 |
| 4 | 1500 | 22.7 | 32.1 | 29.8 | 22.7 |
| 5 | 2000 | 35.1 | 43.2 | 29.5 | 27.1 |
| 6 | 2500 | 44.9 | 56.8 | 27.3 | 27.3 |
| 7 | 3000 | 54.7 | 64.6 | 24.8 | 26.6 |
| 8 | 3500 | 62.2 | 72.9 | 20.2 | 23.8 |
| 9 | 4000 | 69.8 | 79.2 | 18.8 | 22.8 |
| 10 | 4500 | 75.2 | 82.8 | 14.7 | 18.4 |
| 11 | 5000 | 80.0 | 87.8 | 10.6 | 14.2 |
| 12 | 5500 | 84.3 | 89.7 | 9.7 | 13.7 |
| 13 | 6000 | 87.4 | 92.5 | 9.4 | 12.6 |
| 14 | 6500 | 90.3 | 94.4 | 7.8 | 10.5 |
| 15 | 7000 | 93.2 | 96.1 | 6.1 | 8.4 |
| 16 | 7500 | 95.7 | 97.6 | 4.3 | 6.1 |
| 17 | 8000 | 96.5 | 97.9 | 2.5 | 4.4 |
| 18 | 8500 | 97.2 | 98.8 | 2.2 | 4.0 |
| 19 | 9000 | 98.1 | 99.2 | 1.8 | 3.6 |
| 20 | 9500 | 98.5 | 99.6 | 1.6 | 3.4 |
| 21 | 10000 | 98.9 | 99.8 | 1.4 | 3.2 |
| Root mean square error  (g*_i_*/L) | *RMSE* | $\sqrt{\frac{\left(c_{HMF,DM}-c_{HMF,GS}\right)^{2}}{21}}$ | | $\sqrt{\frac{\left(c_{G,DM}-c_{G,W}\right)^{2}}{21}}$ | |
|  |  | 6.50 | | 5.26 | |
| Mean concentration  (g*_i_*/L) | $c_{i,m}=\frac{\sum_{k=1}^{21} c_{i}}{21}$ | *c*_1_,*_HMF,m_* | *c*_2_,*_HMF,m_* | *c*_1_,*_G,m_* | *c*_2_,*_G,m_* |
|  |  | 66.59 | 71.86 | 12.83 | 12.92 |
|  | $\overline{c_{i,m}}=\frac{c_{1,i,m}+c_{2,i,m}}{2}$ | 69.22 | | 12.88 | |
| Coefficient of variation | $CV=\frac{RMSE}{\overline{c_{i,m}}}$ | 0.094 | | 0.408 | |
